# Longitudinal associations between cognitive functioning and depressive symptoms among couples in the Mexican Health and Aging Study

**DOI:** 10.1017/S1041610222000898

**Published:** 2023-01-05

**Authors:** Joan K. Monin, Gail McAvay, Katie Newkirk, Rafael Samper-Ternent

**Affiliations:** 1 Social and Behavioral Sciences Department, Yale School of Public Health, New Haven, CT, USA; 2 Department of Internal Medicine, Yale School of Medicine, New Haven, CT, USA; 3 School of Public Health, The University of Texas Health Science Center, Houston, TX, USA

**Keywords:** cognitive functioning, depression, dyadic analysis, marriage

## Abstract

**Objective:**

To examine the bidirectional associations between older adult spouses’ cognitive functioning and depressive symptoms over time and replicate previous findings from the United States (US) in Mexico.

**Design:**

Longitudinal, dyadic path analysis with the actor-partner interdependence model.

**Setting:**

Data were from the three most recent interview waves (2012, 2015, and 2018) of the Mexican Health and Aging Study (MHAS), a longitudinal national study of adults aged 50 + years in Mexico.

**Participants:**

Husbands and wives from 905 community-dwelling married couples (N = 1,810).

**Measurements:**

The MHAS cognitive battery measured cognitive function. Depressive symptoms were assessed using a modified nine-item Center for Epidemiologic Studies Depression Scale. Baseline covariates included age, education, number of children, limitation with any activity of daily living, limitation with any instrumental activity of daily living, and pain.

**Results:**

As hypothesized, there were significant within-individual associations in which one person’s own cognitive functioning and own depressive symptoms predicted their own follow-up cognitive functioning and depressive symptoms, respectively. In addition, a person’s own cognitive functioning predicted their own depressive symptoms, and a person’s own depressive symptoms predicted their own cognitive functioning over time. As hypothesized, there was a significant partner association such that one person’s depressive symptoms predicted more depressive symptoms in the partner.

**Conclusion:**

Findings from this study of older Mexican couples replicates findings from studies of older couples in the US, showing that depressive symptoms in one partner predict depressive symptoms in the other partner over time; however, there was no evidence for cognition–depression partner associations over time.

## Introduction

As the world’s population of older adults increases, preserving or enhancing cognitive and mental health in late life becomes more important than ever before. There is now a substantial literature showing that the cognitive and mental health of older adults are reciprocally related in nonclinical populations (Perini et al., 2019). Our research and others have found that poorer cognitive functioning is related to greater subsequent depressive symptoms in relatively healthy older adults of different cultural groups (e.g. Black and Hispanic) in the US longitudinally (Monin et al, 2018; Perrino et al., 2008). Research has also consistently shown that older adults who experience greater depressive symptoms concurrently perform poorly on cognitive performance domains, such as episodic memory, information processing, executive functioning, and global cognitive functioning (Bierman, et al, 2005; Butters et al., 2004). Cognitive function and emotions are related and share biological pathways (Linnemann et al., 2020). However, the temporal relationship between changes in cognitive function and emotional changes, operationalized as depressive symptoms, is not completely understood. Because stress processes do not occur for individuals alone, we argue it is important to consider the associations between cognitive functioning and depressive symptoms in the context of ongoing, healthy, older adult, and close relationships.

For married older adults in particular, spouses’ health, stress, and well-being are intertwined (Kiecolt-Glaser and Wilson, 2017). As adults grow older, they tend to constrict their social networks, transitioning from meeting new friends and socializing with acquaintances, to spending more time with close family members and friends (Carstensen et al., 2003). That means, for married older adults, the spousal relationship becomes more central to one’s social environment. Spouses also share many objective environmental and behavioral exposures. They often cohabitate, eat similar foods, do similar physical and leisure activities, share children and family relations, have similar personalities, and they are likely to experience shared stressors (Kiecolt-Glaser and Wilson, 2017). Importantly, in close relationships, one partner’s emotions directly impact the other partner’s emotions for many reasons, including but not limited to empathy, mimicry, and behavioral conditioning (Monin and Schulz, 2009). Multiple dyadic frameworks show how stress is transferred from one partner to the other and are dynamically intertwined. Most commonly cited is Thibault and Kelly’s (1959) interdependence theory which argues that partners mutually influence each other’s outcomes through cognition, emotion, and behavior. Another more recent framework is the Dyadic Biobehavioral Stress Model proposed by Shrout (2021) that outlines the multiple pathways through which coping with a stressor (that is either internal or external from the marriage) is managed by each individual and influences each partner’s health outcomes through psychological, behavioral, and biological pathways. Furthermore, Shrout outlines contextual factors that influence these processes such as each partner’s individual characteristics (e.g. race and gender), illness diagnoses, attachment, life adversity as well as characteristics of the relationship (e.g. socioeconomic status of the couple and relationship length).

There are now multiple studies that have examined older adult, spousal, and reciprocal influences in cognitive functioning and depressive symptoms over time (Gerstorf et al., 2009; Lee et al., 2012; Monin et al., 2018). For example, in a recent study of older adult couples in the US over a span of 7 years in the Cardiovascular Health Study (CHS; Monin et al., 2018), we used the Modified Mini-Mental State Exam (3MS; Bland and Newman, 2001), a clinical measure that assesses a variety of cognitive domains, and the Center for Epidemiological Disease Depression Scale (CESD; Radloff, 1977) to examine the reciprocal partner influences of husbands’ and wives’ cognitive functioning and depressive symptoms. We found a cross-partner effect, such that one spouse’s greater depressive symptoms predicted the other spouse’s lower cognitive functioning, but a spouse’s lower cognitive functioning did not predict the other spouse’s greater depressive symptoms over time. Furthermore, these effects did not differ for husbands and wives. Our study added to a growing body of dyadic studies looking at partner associations cognitive decline and emotional changes; however, no research to our knowledge has examined these dyadic associations in Mexico.

Mexico presents a unique opportunity to examine these dyadic associations because it is “a country experiencing rapid aging occurring alongside historically limited institutional support for older adults, and where traditional gender roles extend to marriage” (p176, Saenz, 2021). It is also important to understand that social relationships are influenced by cultural and structural factors, and we cannot assume that interpersonal processes are the same across different countries. There are specific aspects of Mexican culture that we need to consider when it comes to marriage (Saenz, 2021). First, most older adults in Mexico are married. Among adults ages 50–59 years and 60 + years in Mexico, 76 and 64% were married or in a consensual union as of 2012 (Wong et al., 2017). It is normative to be married as an older adult, and this likely has mental health implications. Also, compared to other Organization for Economic Cooperation and Development (OECD) countries, Mexico has lower rates of divorce and an earlier mean age at first marriage (OECD, 2019). Spouses in Mexico may have a stronger effect on their partner’s mental health than spouses in the US because of a longer period living together. The importance of family may be even more crucial because Mexico lacks adequate institutional support systems for older adults (Robledo et al., 2012). Older adults rely on their family for support more so than in other countries (Peek et al., 2012).

### The present study

An important study provides the opportunity to replicate findings from older adult couple studies of cognitive functioning and depressive symptoms in the US to the Mexican context (Wong et al., 2017). The Mexican Health and Aging Study (MHAS) was designed to examine the influence of disease on the health, function, and life span of adults age 50 + years in Mexico. In the MHAS, data were collected from households, and data were linked for spouses when they both agreed to participate in the study. Included in their comprehensive list of measures was cognitive functioning and depressive symptoms.

The aim of the present study was to examine the reciprocal associations between cognitive functioning and depressive symptoms in the MHAS sample of older adult husbands and wives over time. First, we hypothesized that within each individual lower cognitive functioning would predict future decreases in cognitive functioning, and greater depressive symptoms would predict increases in depressive symptoms over time (H1a and b; actor effects). Second, we hypothesized that one spouse’s lower cognitive functioning would predict decreases in the other spouse’s cognitive functioning and one spouse’s greater depressive symptoms would predict increases in the other spouse’s depressive symptoms over time (H2a and b; partner effects). These hypotheses sought to replicate findings from previous longitudinal studies showing that husbands’ cognitive decline predicts wives’ cognitive decline (Gerstorf et al., 2009) and many studies that show that one partner’s depressive symptoms predict the other partner’s depressive symptoms (e.g. Kouros and Cummings, 2010; Monin et al., 2018) as well as further test multiple theories of interpersonal stress in a Mexican sample. Third, we hypothesized that within individuals there would be an association where one’s poorer cognitive functioning predicts one’s higher number of depressive symptoms over time (H3; cross-lagged actor effects). Fourth, we hypothesized an association where an individual’s poorer cognitive functioning predicts a higher number of the spouse’s depressive symptoms over time (H4; cross-lagged partner effects). Finally, we explored whether there were gender differences for partner effects concerning whose cognitive functioning and depressive symptoms were more predictive of their partner’s cognitive functioning and depressive symptoms. Some research has shown that husbands’ depressive symptoms predict wives’ depressive symptoms (e.g. Tower & Kasl, 1996). It has been argued this is due to women’s societal role to focus more on controlling or facilitating the well-being of others in close relationships compared to men (Cross and Madson, 1997). Also, among older adults couples, wives tend to be the caregivers, with caregivers showing more emotional distress in response to their partner’s suffering (Monin et al., 2019). However, other studies show that wives’ depressive symptoms affect husbands’ depressive symptoms more strongly (e.g. Thomeer et al., 2013). Our past findings regarding the present hypotheses in a US sample show no gender effects (Monin et al., 2018). Thus, there is still a need to explore gender for these interpersonal effects, and in a new cultural context.

## Method

### Participants

The data for this study are from the MHAS, a longitudinal national study of adults aged 50 years and older residing in Mexico (Samper-Ternent et al., 2012; Wong & Palloni, 2009; Wong et al., 2017). The first survey was conducted in 2001, with follow-ups in 2003, 2012, 2015, and 2018. The current study focuses on the more recent data from the three interviews conducted in 2012, 2015, and 2018. Individuals who had two direct interviews and whose spouse was also directly interviewed in 2012 were included in the analysis sample. We also restricted the sample by age, including participants aged 65 years and older. Both spouses were aged 65 years and older. We identified, 1,810 individuals, consisting of 905 couples who had direct interviews in 2012 and at least one follow-up direct interview in 2015 or 2018. Of these, 1,774 (98%) had a direct interview in 2015, and 1,389 (76.7%) completed the 2018 assessment.

### Outcomes

#### Cognitive function

The MHAS cognitive battery was administered during the direct interviews to measure cognitive function. This instrument was chosen to avoid bias in measuring cognition for individuals with limited literacy and mathematical ability (Michaels-Obregón et al., 2022). The MHAS Cognitive battery was initially derived from the Cross-Cultural Cognitive Evaluation (CCCE) developed by Glosser and colleagues in 1993. As mentioned in the document provided by the MHAS Workgroup on their webpage (Michaels-Obregon et al., 2022) all questions were translated, back-translated, and culturally adapted to the Mexican populations by a group of experts in Mexico. Several cognitive tasks have been added to include a more comprehensive cognitive evaluation and improve the sensitivity of the cognitive battery. The MHAS cognitive battery was validated in a clinical convenience cohort and in a population-based cohort before implementation in the MHAS initial wave in 2001. In the last 20 years, multiple studies have shown validity and reliability of this cognitive battery. More detailed information is available in the MHAS website (http://mhasweb.org/Resources/DOCUMENTS/Constructed_Imputed/MHAS_Cognitive_Function_Measures_Scoring_and_Classification.pdf). The MHAS cognitive battery assesses several domains of cognition, and we focused on the seven cognitive tasks that were measured at each of the 2012–2018 interviews.

*Immediate memory* was measured by having the subject repeat eight words and was scored from 0 to 8 words repeated. *Delayed memory* was then assessed later in the interview where the subject was asked to recall words from the earlier list, scored from 0 to 8 words recalled. *Visual scanning* involved the respondent identifying as many animals as possible within 1 minute and was scored 0–60. *Constructional Praxis*, a measure of executive function, was assessed by asking the respondent to draw two geometrical figures within 90 seconds. The score ranges from 0 to6 reflecting the degree to which the figures were copied. *Constructional Praxis Recall* was assessed later in the interview by asking respondents to redraw the same figures given earlier by recall and ranged from 0 to 6. *Verbal fluency* was measured by asking respondents to name as many figures as they can in 60 seconds, scored 0 to 60. *Orientation* was assessed by asking the participant to recall the day, month, and year, scored 0–3 to reflect the number correctly identified (Michaels-Obregón et al., 2014). “The average correlation between the seven cognitive tasks was 0.31 for husbands and 0.30 for wives, with standard deviations of 0.10 and 0.11, respectively. (See Supplemental Table 1 for details).”

Because the ranges for the seven cognitive domain scores varied, we rescaled each by dividing the raw score by the maximum possible value for each subscale (Seeman et al., 1994). The rescaled subscale scores thus range from 0 (worst) to 1 (best) and represent the proportion of the best score that the individual achieved. The final total cognitive score was created by taking the mean of the seven subscales. We also considered standardizing each subscale, but not all subscales had a normal distribution, making it difficult to interpret the scores.

#### Depressive symptoms

Self-reported depressive symptoms were assessed using a modified Center for Epidemiologic Studies Depression Scale (CESD; Radloff, 1977). The reliability and validity of this modified scale have been established in this population (Aguilar-Navarro et al, 2007). The presence of nine symptoms (1 = experienced versus 0 = did not experience) in the previous week was assessed for 1) feeling depressed, 2) feeling everything was an effort, 3) restless sleep, 4) feeling happy (reverse scored), 5) feeling lonely, 6) enjoyed life (reverse scored), 7) feeling sad, 8) feeling tired, and 9) having a lot of energy (reverse-scored).

#### Covariates

Sociodemographic characteristics included age, sex, language (Spanish vs. English), number of children, employment status, and years of education (Wong, 2013; Wong et al., 2017). Two measures of disability were created by separately summing difficulties with activities of daily living (ADL) and difficulties with instrumental activities of daily living (IADL). For ADLs, these included bathing, eating, transfer from bed, toileting, and dressing, and for IADLs we included preparing meals, shopping, managing medications, and managing money. If the respondent did not do the activity but received help for the activity, then the item was coded as disabled (Díaz-Venegas et al., 2018). If the respondent said they had difficulty doing the activity or that they could not do the activity, the item was also coded as disabled. Comorbidity was measured by summing the self-report of a physician diagnosis for the following conditions: hypertension, diabetes, cancer, chronic lung disease, myocardial infarction, stroke, arthritis, liver, and kidney disease. Finally, smoking was coded as 1 = current versus 0 = not current smoker and the pain question was coded yes = 1 versus No = 0.

### Statistical analysis

Baseline characteristics were summarized for husbands and wives separately, as frequencies and percentages for categorical variables or means and standard errors for the continuous measures. Correlations within and between husbands and wives were estimated for cognition and depressive symptoms, and the associations between the baseline covariates and cognition and depressive symptoms were also examined.

We first estimated the change from baseline in cognitive function and depression by using a generalized estimating equations (GEEs) approach that included both husbands and wives simultaneously and each interview (time) for the two outcomes. The dependencies in the data due to the correlation between members of the same couple and the correlation over time induced by the repeated measures on each person were modeled by specifying an unstructured working correlation matrix. The model we estimated included two intercepts to reflect the husband’s and wife’s level of the outcome in 2012 (intercept), and two indicator variables for each member of the couple, that reflected the year of the interview (2015 and 2018). These terms provide contrasts between the scores in 2015 and 2018 with the baseline (2012) score which was the referent time point. The change from baseline in cognition and depression was estimated using the coefficients for these terms. The predicted marginal means were then computed to add to the interpretation of change. This categorical coding of time was necessary, since the relationship between time and the outcomes was not linear.

In our second analysis, a three-wave, crossed-lag actor-partner interdependence structural equation model (SEM) with robust standard errors was estimated to test our hypotheses. To examine the directionality of the association between cognitive functioning and depression, we estimated two SEM models. The first model focused on estimating the effects of cognitive functioning on depressive symptoms as the outcome, while the second model estimated the effects of depression on cognitive functioning. We began with a saturated model, where separate actor and partner effects and separate time effects were estimated for each follow-up period. We then tested a model where the associations over time were constrained to be equal, specifically T1 to T2 and T2 to T3 were set to be equal. Following this, we next examined gender effects, by comparing the fit of models where the actor and partner effects for husbands and wives were constrained to be equal, to a model with separate effects.

Baseline covariates measured in 2012 included age, education, number of children, ADL limitations, IADL limitations, and pain as predictors of the two outcomes in 2015 and 2018, as these were the variables with correlations >= 0.20 with depression or cognition. For the error terms at the same time point, we estimated the covariance to account for residual nonindependence in the outcomes. We calculated Wald test statistics to test the model constraints, since chi-square difference tests are not valid when analyzing imputed datasets. In addition, model fit was assessed by using the comparative fit index (CFI), Tucker–Lewis indices (TLI), and the root mean square error (RMSEA) for each model. Values of greater than 0.95 for the CFI, values greater than 0.90 for the TLI, and values less than 0.08 for the RMSEA reflect a good fit of the model to the data (7).

Missing data in the direct interviews, missing data due to nonrespondents, and missing data due to death occurred primarily on the cognitive measures (Immediate memory, 10.1 %; Delayed Memory 10.2%, Visual scan 19.1%; Constructional Praxis 18.5%; Constructional Praxis Recall 20.9%; Verbal Fluency 10.5%; and Orientation 9.4%), but there was also intermittent missing data on depression (9.3%) and the covariates (<= 9.6%). Missingness in cognitive measures has been reported in similar population-based studies. Studies using MHAS data have reported that visual impairment, inability to hold a pencil to complete the paper-based assessment, and refusal to complete the cognitive section are the most commons reasons for missing cognitive data. We used multiple imputation methods, based on fully conditional specifications, assuming the data were missing at random (MAR). Previous studies using MHAS data, although not dyadic, have used similar approaches for missing data (Downer et al., 2021). We used an extensive number of variables from 2012 to 2018 in the imputation process including measures of sociodemographic characteristics, self-rated health, chronic conditions, mobility difficulties, ADL and IADL, vision and hearing, pain, self-reported memory, physical symp toms, smoking, employment status, finances, life satisfaction, activity level, social support, cognitive subscales, and depression items. We examined the relationships between these variables and missingness in cognitive functioning and depression and their association with death.

Using SAS/STAT version 9.4 software, 20 datasets were imputed using the SAS procedure PROC MI. Models were fit for each of the multiple datasets generated from the imputation, and estimates were combined using Rubin’s rules as implemented in the SAS software procedure Proc MIANALYZE and in MPLUS Software. For the main analysis, we used the imputed data for participants with at least two interviews, excluding decedents. To evaluate the potential bias due to missingness and the competing risk of death, we performed sensitivity analyses for the two outcomes. First, we estimated a complete case model (not imputed), and then three models assuming different scenarios in which the data are missing not at random (MNAR) for those who were deceased. Specifically, we repeated the main analysis three times by setting the values of the cognitive and depression outcomes 1) to the mean value, 2) to one standard deviation below the mean, and 3) to one standard deviation above the mean separately for husbands and wives.

## Results

The baseline (2012) characteristics of the sample are presented in Table 1. On average, husbands were older than wives, 73.4 years versus 70.6 years, and men had more years of education than women, 4.6 years versus 3.9 years, respectively. English was rarely spoken by couples, only 3.4% for men and 1.5% for women, and couples had an average of 5.9 children. Husbands were more likely to be employed, 35.0% of men had jobs, while 10.4% of women worked. Smoking was reported by 133 (14.7%) men, while only 31 (3.4%) women smoked. Comorbidity, specifically having two or more conditions, was higher in women (39.1%) than men (27.1%), and wives were also more likely to report an ADL (22.1%) or IADL (15.0%) difficulty than their husbands (ADL = 17.9% and IADL = 9.4%). Almost half (45.0%) of the wives reported pain, and a substantial number of husbands also reported having pain (36.9%).

The correlation of cognitive functioning across time was approximately 0.65 for both men and women (Table 2), while the associations between the couples’ cognitive scores were lower but still substantial, ranging from a low of 0.25 to a high of 0.40. The correlation of depression across interviews was lower than that for cognitive function, ranging from 0.30 to 0.50, for men and women, while the association between husbands’ and wives’ depression ranged from 0.11 to 0.28.

As expected, older age (− 0.28 to − 0.37) was negatively associated with cognitive functioning, while higher education (0.43 to .053) was associated with better cognitive functioning scores for both husbands and wives. Having more children (− 0.23 to − 0.27) was also associated with poorer cognitive functioning (Table 3). For depression, number of health conditions (0.11 to 0.30), ADL limitations (0.16 to 0.32), IADL limitations (0.17 to 0.25), and pain (0.16 to 0.41) had the largest correlations. Results of the GEE model are displayed in Table 4. There was a significant decline in cognitive functioning from the baseline (2012) to the 2^nd^ (2015) and 3^rd^ (2018) follow-up interviews, for both husbands and wives. However, the means from 2015 to 2018 did not differ for either member of the couple. For husbands, the predicted means ranged from 62.8 to 59.3, while for women the range was 62.8 to 60.2. Table 4 also presents the increase in depressive symptoms at each follow-up interview relative to that baseline. Both husbands and wives had significantly more symptoms over time, with the predicted mean ranging from of 2.8 to 3.1 for husbands and 3.7 to 3.9 for wives.

### SEM results

To begin the SEM modeling analyses, we first estimated two saturated models with separate effects for each follow-up period (i.e. time) and for each gender, to be used as a baseline model. The Wald statistic for equal time constraints was not significant for both Model 1 with cognitive functioning predicting depression (Wald statistic = 9.78, df = 11, *p*-value = 0.55) or Model 2 with depression predicting cognitive functioning (Wald statistic = 13.326, df = 11, *p*-value = 0.27). Therefore, we next fit a model assuming both equal time and equal gender effects. The Wald statistics for both Model 1 (Wald statistic = 9.59, df = 11; *p*-value = 0.14) and Model 2 (Wald statistic = 8.06, df = 6, *p*-value = .23) were not significant, so the final models estimated had equal time and equal gender effects. This model is depicted in Supplemental Figure 1, where paths with the same lowercase letter are those constrained to be equal. The estimated path coefficients for the final models are presented in Table 5A and B. The model fit was good for both cognitive functioning (Model 1: CFI of 0.95, TLI of 0.91 and RMSEA of .05) and depression (Model 2: CFI of 0.95, TLI of 0.91 and RMSEA of .05) (Hu & Bentler, 1999). The average r-squared across time for cognitive functioning was 0.48, while the r-squared for depression was 0.23.

There were significant actor associations for both cognitive functioning and depression, with a person’s own cognitive functioning (hypothesis 1a actor effects) and their own depression (Hypothesis 1b actor effects), predicting their own follow-up assessment of cognition and depression. For hypothesis 2a (partner effects), there was not a significant association between one’s own cognitive functioning influencing one’s partner cognitive functioning. However, hypothesis 2b was supported in that there was a partner association for depression, in which one’s own depression was associated with more depressive symptoms in the spouse. For hypothesis 3, a person’s own cognitive functioning predicted their own depressive symptoms (Model 1 actor cross-lagged effect) and one’s own depressive symptoms predicted their own cognitive functioning across time (Model 2 actor cross-lagged effect). Hypothesis 4, that proposed a significant cross-lagged partner effect, was not supported for Model 1, where one’s own cognitive function was hypothesized to influence one’s spouse’s depressive symptoms, or for Model 2 in which one’s own depressive symptoms did not have a significant association with their spouse’s cognitive functioning across time.

To examine the magnitude of the effects, we reran the model with z-scores to determine the standardized effects (column labeled Beta in Table 5A and B). Using the standard deviation of 14.2 for cognitive functioning and the standard deviation of 2.58 for depressive symptoms, the effect size column (ES) shown in Table 5A and B reflects the effect of a one standard deviation change in the predictor variable on one standard deviation of the outcome variable. For example, the effect size for the actor effect of cognitive functioning on depression under Model 1 was Beta = − 0.054 multiplied by 2.58 = − 0.14. This indicates that for one standard deviation decrease in one’s own cognitive functioning, there is an increase of 0.14 in one’s own depressive symptoms. The effect size for the partner effect of depression under Model 1 was Beta = 0.039 multiplied by 2.58 = 0.10, which indicates that an increase in one standard deviation in one’s own depression, is associated with an increase of 0.10 in one’s partner’s depressive symptoms. The effect sizes are small and may not be clinically meaningful on their own. However, it is still important to document statistically significant findings such as these, as they may combine with other small effects that lead to more meaningful changes in depressive symptoms. The effects may have been larger if the time of follow-up was shorter, for example, a year instead of 3 years. A number of events could have occurred over 3 years that led to different changes in cognition and/or depression prior to the end of the 3-year interval.

We estimated the association of 22 study variables with missingness in the cognitive and depression measures across the three waves of the study. Age, education, number of children, self-rated health, mobility limitations, ADLs, IADLs, vision, physical symptoms, employment status, life satisfaction, and activities were all associated with missing cognitive function on at least two study waves (Supplemental Table 2). Preceding and concurrent depression and preceding cognition also predicted missingness in cognition. There were fewer associations with missing depressive symptoms, specifically age, chronic conditions, wearing a hearing aid, physical symptoms, and preceding cognitive function were associated with missing depressive symptoms on at least one study wave (Supplemental Table 3). The majority of the 22 study variables were associated with death, as shown in Supplemental Table 4. Given these associations, it was important to conduct sensitivity analyses.

In the sensitivity analyses, we estimated models under three scenarios: a complete case analysis (not imputed), setting missing to the mean, and setting missing to one standard deviation above the mean and one standard deviation below the mean. The results of these analyses are presented in the Supplemental Table 5 and Table 6. As seen in the primary analyses without deaths, Hypotheses 1a and 1b, actor effects for cognition and depression were supported across all scenarios for both Model 1 and Model 2. In addition, the results for hypothesis 2a, agreed with the findings when excluding deaths, with no significant partner effects for cognition predicting one’s spouses cognitive functioning. For the most part, hypothesis 2b, hypothesizing partner effects on depression followed the original analyses, for Model 2, while for Model 1 the associations were marginal (*p* < 0.10). Finally, the results for hypotheses 3 and 4 supported the original findings, except for hypothesis 3, Model 2, the associations were marginal when assigning poorer outcomes to the data missing due to death.

## Discussion

In this study, we found both actor and partner effects for the associations between cognitive functioning and depressive symptoms among older couples in Mexico. First, we found that an individual’s own greater cognitive functioning predicted their own greater cognitive functioning in the future (hypothesis 1a). Second, an individual’s greater depressive symptoms predicted their own greater depressive symptoms in the future (hypothesis 1b). Third, an individual’s greater depressive symptoms predicted their spouse’s greater depressive symptoms in the future (hypothesis 2b); this was not the case for cognitive functioning (1a). Fourth, an individual’s own lower cognitive functioning predicted their own greater depressive symptoms in the future, and an individual’s own greater depressive symptoms predicted their own lower cognitive functioning in the future (hypothesis 3). There were no cross-partner associations for cognitive functioning and depressive symptoms (hypothesis 4). In the final model, gender effects were constrained to be equal, suggesting a similar pattern of associations for husbands and wives for all effects.

The within-person associations between cognitive functioning and depressive symptoms in both directions are consistent with previous research (Monin et al., 2018), and the present findings extend past findings to older adults in the Mexican population. Further, they add to a growing literature that suggests that cognitive functioning and depressive symptoms have shared and/or overlapping pathways for older adults. There is a recent framework that recognizes common etiological factors like age and vascular changes in the brain that lead to sensory deficits that may lead to cognitive impairment through depression and that also lead directly to higher risk of depression (Whitson et al., 2018). In their model, Whitson and colleagues propose that sensory impairments can lead to cognitive load, brain structure changes, depression, social isolation, and reduced activity, which can in turn lead to impaired cognitive functioning. More work is needed to understand these psychosocial and biological mechanisms for the links between depressive symptoms and cognitive decline in older adults. For example, changes in sensory impairments may be a mechanism to examine in future studies of older adult spouses, as sensory impairments may affect communication within spousal relationships in protective or harmful ways.

This study’s significant partner effects of depressive symptoms are not surprising. Although the effect sizes are small, they support a large literature showing that greater depressive symptoms in one spouse increases depressive symptoms in the other spouse over time (see Monin and Schulz, 2009 for review). This finding has been shown in many contexts across relationships across the lifespan, in different cultures (Liu et al., 2022), and it is consistent with theories of emotion contagion (Hatfield et al., 1993), attachment theory (Bowlby, 1969), and the linked lives framework (Kiecolt-Glaser and Wilson, 2017), to name a few. However, there was no evidence of gender-specific findings for the partner effects, where wives are more influenced by their husbands than vice versa (Cross and Madson, 1997).

Unlike in our CHS analysis (Monin et al., 2018), we did not find cross-partner associations between cognitive functioning and depressive symptoms in the Mexican population. Recall in the CHS study, we found that having a spouse who is depressed increases a person’s risk for cognitive decline over time. It may be the case that in this Mexican population, there are different spousal behavioral dynamics or cultural influences that make it less likely for one spouses’ cognition or depression to influence the other spouse. Related to this, Fuller-Iglesias and Antonucci (2016) found that in Mexico, older adults had larger, more geographically proximate networks with a greater proportion of kin but less frequent contact than younger adults. It may be that the social network does not narrow to the extent that it does for older adults in US population, which could protect spouses from partner effects on cognitive decline. Alternatively, the present study findings may be a consequence of using a different measure of cognitive functioning. Yet another explanation is that the partner effect for cognition that we found in the CHS dataset was specific to that study, underscoring the importance of replicating effects across different populations and with different measures to examine how robust the phenomenon is.

This study has many strengths. First is the inclusion of linked spouses in the dataset. Second is the large, representative sample from Mexico. Third is the extensive number of health-related and psychosocial variables we could include as potential covariates. Fourth, the longitudinal design allowed us to examine change over time.

In summary, this study adds to a large literature showing that older adult spouses’ influence one another’s health in terms of depressive symptoms. We also found further evidence that within individuals, cognitive functioning and depressive symptoms are highly related in later life. This study extends past research by showing that the partner effects of depressive symptoms and the within-person effects of cognitive function and depressive symptoms are not limited to one cultural context. Mexican older adult spouses experience similar interpersonal emotional influences. However, future research is needed to understand spousal behaviors in Mexico to more fully understand how we can support older couples to thrive and stay resilient if one partner becomes depressed.

## Supplementary Material

1

## Figures and Tables

**Table 1.** Baseline (2012) characteristics of MHAS participants

	Husbands (*N* = 905)	Wives (*N* = 905)

	Mean (SE)	Mean (SE)
Age in years:	73.4 (0.18)	70.6 (0.16)
Years of education:	4.6 (0.15)	3.9 (0.12)
English speaking: n (%)	31 (3.4)	14 (1.5)
Number of children:	5.9 (0.10)	5.9 (0.10)
Currently employed: n (%)	317 (35.0)	94 (10.4)
Current smoker: n (%)	133 (14.7)	31 (3.4)
Conditions: n (%)		
0	352 (38.9)	221 (24.4)
1	308 (34.0)	330 (36.5)
2 or more	245 (27.1)	354 (39.1)
Pain: n (%)	334 (36.9)	407 (45.0)
ADLs: n (%)		
0	743 (82.1)	705 (77.9)
1	111 (12.2)	128 (14.1)
2 or more	51 (5.7)	72 (8.0)
IADLs: n (%)		
0	820 (90.6)	769 (85.0)
1	50 (5.5)	98 (10.8)
2 or More	35 (3.9)	38 (4.2)

Note: SE = Standard error of mean.

**Table 2.** Pairwise correlations of husbands (H) and wives (W) cognitive functioning (cog) and depressive symptoms (dep)

	Hcog2012	Hcog2015	Hcog2018	Wcog2012	Wcog2015	Wcog2018

Hcog2012	–					
Hcog2015	0.65	–				
Hcog2018	0.58	0.63	–			
Wcog2012	0.40	0.33	0.33	–		
Wcog2015	0.29	0.32	0.31	0.67	–	
Wcog2018	0.25	0.28	0.33	0.56	0.65	–
	Hdep2012	Hdep2015	Hdep2018	Wdep2012	Wdep2015	Wdep2018
Hcog2012	–					
Hcog2015	0.45	–				
Hcog2018	0.30	0.36	–			
Wcog2012	0.24	0.19	0.10	–		
Wcog2015	0.18	0.28	0.11	0.50	–	
Wcog2018	0.11	0.21	0.17	0.44	0.45	–

**Table 3.** Pairwise correlations for husbands (H) and wives (W) covariates at baseline with cognitive functioning (cog) and depressive symptoms (dep) in 2012, 2015, 2018

	Hcog2012	Hcog2015	Hcog2018	Wcog2012	Wcog2015	Wcog2018

Husbands baseline:							
Age	−0.30	−0.37	−0.38	−0.28	−0.36	−0.33
Years of education	0.46	0.49	0.43	0.53	0.49	0.47
Number of children	−0.23	−0.27	−0.27	−0.26	−0.23	−0.23
Conditions	0.01	0.01	0.01	−0.03	−0.04	−0.04
ADLs	−0.10	−0.11	−0.07	−0.09	−0.08	−0.05
IADLs	−0.18	−0.17	−0.13	−0.13	−0.12	−0.07
Employed	0.10	0.11	0.03	0.07	0.08	0.06
Current smoker	−0.04	0.02	0.03	0.05	0.07	0.02
Pain	−0.03	−0.02	−0.05	−0.02	0.01	0.02
Finances (1=excellent to 5=poor)	−0.16	−0.13	−0.12	−0.14	−0.10	−0.12
	Hdep2012	Hdep2015	Hdep2018	Wdep2012	Wdep2015	Wdep2018
Wives baseline:						
Age	0.03	0.06	0.05	0.03	0.08	0.09
Years of education	−0.18	−0.21	−0.20	−0.23	−0.17	−0.23
Number of children	0.07	0.11	0.09	0.07	0.07	0.10
Conditions	0.25	0.15	0.11	0.30	0.23	0.20
ADLs	0.31	0.24	0.16	0.32	0.24	0.20
IADLs	0.21	0.17	0.07	0.25	0.18	0.12
Employed	−0.09	−0.11	−0.07	−0.06	−0.05	−0.04
Current smoker	−0.01	0.04	0.01	−0.03	0.01	0.01
Pain	0.41	0.31	0.16	0.37	0.30	0.23
Finances (1=excellent to 5=poor)	0.24	0.22	0.17	0.28	0.23	0.21

**Table 4.** Husbands’ and wives’ cognitive function and depressive symptoms over time

	Cognitive functioning			Depressive symptoms		

Predicted mean (SE):	2012	2015	2018	2012	2015	2018
Husbands	62.8 (0.49)	59.6 (0.52)	59.3 (0.59)	2.8 (0.08)	3.1 (0.08)	3.1 (0.09)
Wives	62.8 (0.48)	60.9 (0.52)	60.2 (0.54)	3.7 (0.09)	3.9 (0.09)	3.9 (0.10)
Change from 2012:		β (SE)	β (SE)		β (SE)	β (SE)
Husbands	–	− 3.3 (0.44)***	− 3.6 (0.53)***	–	0.23 (0.08)***	0.24 (0.10)*
Wives	–	− 2.0 (0.42)***	− 2.7 (0.50)***	–	0.18 (0.09)*	0.22 (0.11)*

Note: SE = Standard error; Predicted mean is obtained from a generalized estimating equation (GEE) model.

* *p* < .05

** *p* < .01

*** *p* < 0.002.

**Table 5.** Estimates from structural equation models: hypothesized actor and partner effects

A. Model 1, in which paths from cognitive functioning to depression are included					

	B	SE	*P*	Beta	ES

Hypothesis 1a (actor): Cog → Cog	0.503	0.021	<0.001	0.491	6.97
Hypothesis 2a (partner): Cog → Partner Cog	0.011	0.017	0.545	0.007	0.10
Hypothesis 1b (actor): Dep → Dep	0.333	0.022	<0.001	0.332	0.86
Hypothesis 2b (partner): Dep →Partner Dep	0.034	0.019	0.074	0.039	0.10
Hypothesis 3 (actor): Dep → Cog	− 0.009	0.004	0.011	− 0.054	− 0.14
Hypothesis 4 (partner): Dep→ Partner Cog	− 0.002	0.004	0.549	− 0.012	− 0.03

B. Model 2, in which paths from depression to cognitive functioning are included					

	β	SE	*P*	Beta	ES

Hypothesis 1a (actor): Cog → Cog	0.496	0.021	<0.001	0.485	6.89
Hypothesis 2a (partner): Cog → Partner Cog	0.006	0.018	0.738	0.003	0.04
Hypothesis 1b (actor): Dep → Dep	0.341	0.022	<0.001	0.340	0.88
Hypothesis 2b (partner): Dep →Partner Dep	0.038	0.019	0.041	0.043	0.11
Hypothesis 3 (actor): Dep → Cog	− 0.193	0.089	0.030	− 0.032	− 0.45
Hypothesis 4 (partner): Dep→ Partner Cog	− 0.102	0.085	0.228	− 0.017	0.24

Notes: Cog = cognitive functioning; Dep = depression; SE = standard error; Beta = standardized coefficient. ES = effect size is the change in outcome for a one standard deviation unit change in the predictor.
