# Supplementary material for: Longitudinal associations between cognitive functioning and depressive symptoms among couples in the Mexican Health and Aging Study
Source: Int Psychogeriatr. Author manuscript; Available in PMC 2024 Apr 1. (PMC10319915; doi:10.1017/S1041610222000898)
Supplement: 1 [file NIHMS1838342-supplement-1.docx]

| S-Table 1. Baseline (2012) Correlations Between Cognitive Tasks for Husbands and Wives | | | | | | | |
| --- | --- | --- | --- | --- | --- | --- | --- |
| HUSBANDS | Constructional-  Praxis  (CP) | Constructional-Praxis Recall  (CPR) | Delayed Memory  (DM) | Immediate Memory  (IM) | Orientation (O) | Verbal  Fluency  (VF) | Visual Scanning  (VS) |
| CP | 1.0 |  |  |  |  |  |  |
| CPR | .52 | 1.0 |  |  |  |  |  |
| DM | .17 | .32 | 1.0 |  |  |  |  |
| IM | .24 | .33 | .56 | 1.0 |  |  |  |
| O | .27 | .30 | .26 | .27 | 1.0 |  |  |
| VF | .19 | .23 | .28 | .41 | .24 | 1.0 |  |
| VS | .30 | .34 | .30 | .43 | .27 | .38 | 1.0 |
| WIVES | Constructional-  Praxis  (CP) | Constructional-Praxis Recall  (CPR) | Delayed Memory  (DM) | Immediate Memory  (IM) | Orientation (O) | Verbal  Fluency  (VF) | Visual Scanning  (VS) |
| CP | 1.0 |  |  |  |  |  |  |
| CPR | .59 | 1.0 |  |  |  |  |  |
| DM | .17 | .30 | 1.0 |  |  |  |  |
| IM | .24 | .27 | .52 | 1.0 |  |  |  |
| O | .28 | .28 | .21 | .25 | 1.0 |  |  |
| VF | .17 | .20 | .25 | .36 | .19 | 1.0 |  |
| VS | .33 | .32 | .34 | .40 | .32 | .39 | 1.0 |

| S-Table 2. Logistic Regression Model Results for Missing Cognitive Functioning Outcome at each Study Wave (Missingness Outcome coded: 1=Missing 0=Not Missing) | | | | | | | | | | | | |
| --- | --- | --- | --- | --- | --- | --- | --- | --- | --- | --- | --- | --- |
|  | 2012 | | | | 2015 | | | | 2018 | | | |
| Variable: | Odds Ratio | 95% CI | | p-value | Odds Ratio | 95% CI | | p-value | Odds Ratio | 95% CI | | p-value |
| Female vs male | 0.86 | 0.66 | 1.13 | 0.273 | 0.90 | 0.69 | 1.18 | 0.448 | 0.91 | 0.74 | 1.12 | 0.390 |
| Age in years | 1.09 | 1.06 | 1.11 | 0.000 | 1.09 | 1.06 | 1.11 | 0.000 | 1.09 | 1.07 | 1.11 | 0.000 |
| Educational level | 0.82 | 0.78 | 0.86 | 0.000 | 0.88 | 0.84 | 0.92 | 0.000 | 0.92 | 0.90 | 0.95 | 0.000 |
| Number living children | 1.08 | 1.03 | 1.13 | 0.001 | 1.08 | 1.04 | 1.13 | 0.001 | 1.08 | 1.04 | 1.12 | 0.000 |
| Self-rated health  (1=Excellent to 5=Poor) | 1.27 | 1.07 | 1.51 | 0.008 | 1.14 | 0.96 | 1.36 | 0.131 | 1.19 | 1.04 | 1.35 | 0.011 |
| Number chronic conditions | 1.00 | 0.88 | 1.14 | 0.989 | 1.11 | 0.99 | 1.26 | 0.085 | 1.05 | 0.95 | 1.15 | 0.375 |
| Number mobility limitations | 1.06 | 1.01 | 1.10 | 0.011 | 1.09 | 1.04 | 1.13 | 0.000 | 1.03 | 1.00 | 1.07 | 0.073 |
| Number ADL limitations | 1.28 | 1.10 | 1.48 | 0.001 | 1.25 | 1.08 | 1.45 | 0.003 | 1.07 | 0.93 | 1.24 | 0.337 |
| Number IADL limitations | 1.71 | 1.43 | 2.03 | 0.000 | 1.89 | 1.59 | 2.26 | 0.000 | 1.24 | 1.02 | 1.50 | 0.031 |
| Wears a Hearing Aid  1=Yes 0=No | 1.28 | 0.48 | 3.37 | 0.621 | 0.99 | 0.34 | 2.87 | 0.988 | 2.28 | 1.03 | 5.03 | 0.041 |
| Vision  (1=Excellent to 5=Poor/Blind) | 1.77 | 1.49 | 2.11 | 0.000 | 1.72 | 1.44 | 2.05 | 0.000 | 1.25 | 1.10 | 1.42 | 0.001 |
| Pain (1=Yes 0=No) | 1.24 | 0.95 | 1.63 | 0.116 | 1.09 | 0.83 | 1.44 | 0.527 | 0.99 | 0.80 | 1.22 | 0.895 |
| Self-rated Memory  (1=Excellent to 5=Poor) | 1.15 | 0.96 | 1.38 | 0.135 | 1.11 | 0.93 | 1.32 | 0.232 | 1.13 | 0.99 | 1.29 | 0.062 |
| Number physical symptoms | 1.10 | 1.01 | 1.20 | 0.035 | 1.11 | 1.02 | 1.22 | 0.020 | 1.04 | 0.97 | 1.12 | 0.278 |
| Smoker (yes vs no) | 0.77 | 0.46 | 1.28 | 0.306 | 0.94 | 0.58 | 1.52 | 0.805 | 0.70 | 0.48 | 1.04 | 0.076 |
| Employed (yes vs. no) | 0.65 | 0.45 | 0.92 | 0.016 | 0.49 | 0.34 | 0.73 | 0.000 | 0.94 | 0.74 | 1.20 | 0.631 |
| Finances  (1=Excellent to 5=Poor) | 1.21 | 0.97 | 1.50 | 0.089 | 1.08 | 0.87 | 1.33 | 0.486 | 1.00 | 0.85 | 1.17 | 0.988 |
| Life is close to ideal:  (1=Agree, 2=Neutral; 3=Disagree) | 1.44 | 1.13 | 1.84 | 0.003 | 1.12 | 0.86 | 1.45 | 0.391 | 1.38 | 1.13 | 1.68 | 0.002 |
| Number activities perform | 0.64 | 0.59 | 0.69 | 0.000 | 0.72 | 0.67 | 0.78 | 0.000 | 0.78 | 0.74 | 0.83 | 0.000 |
| Social Support from spouse  (1=A lot to 3=Not at all) | 1.08 | 0.81 | 1.43 | 0.598 | 1.16 | 0.87 | 1.55 | 0.306 | 1.15 | 0.92 | 1.42 | 0.223 |
| Depression: 2012 | 1.09 | 1.04 | 1.15 | 0.001 | 1.06 | 1.01 | 1.11 | 0.029 | 1.06 | 1.01 | 1.10 | 0.008 |
| Depression 2015 | - | - | - | - | 1.12 | 1.06 | 1.18 | 0.000 | 1.07 | 1.03 | 1.12 | 0.001 |
| Depression 2018 | - | - | - | - | - | - | - | - | 1.10 | 1.05 | 1.16 | 0.000 |
| Cognition: 2012 | - | - | - | - | 0.96 | 0.95 | 0.97 | 0.000 | 0.97 | 0.96 | 0.97 | 0.000 |
| Cognition 2015 | - | - | - | - | - | - | - | - | 0.96 | 0.95 | 0.97 | 0.000 |

| S-Table 3. Logistic Regression Model Results for Missing Depressive Symptoms Outcome at each Study Wave (Missingness Outcome coded: 1=Missing 0=Not Missing) | | | | | | | | |
| --- | --- | --- | --- | --- | --- | --- | --- | --- |
|  | 2015 | | | | 2018 | | | |
| Variable | Odds Ratio | 95% CI | | p-value | Odds Ratio | 95% CI | | p-value |
| Female vs male | 0.86 | 0.50 | 1.48 | 0.581 | 0.95 | 0.73 | 1.23 | 0.684 |
| Age in years | 1.00 | 0.95 | 1.06 | 0.866 | 1.06 | 1.03 | 1.09 | 0.000 |
| Educational level | 1.03 | 0.96 | 1.09 | 0.433 | 1.00 | 0.97 | 1.03 | 0.990 |
| Number living children | 1.07 | 0.98 | 1.17 | 0.150 | 1.03 | 0.98 | 1.08 | 0.219 |
| Self-rated health:  (1=Excellent to 5=Poor) | 1.12 | 0.79 | 1.59 | 0.513 | 0.99 | 0.84 | 1.16 | 0.868 |
| Number chronic conditions | 1.26 | 1.00 | 1.59 | 0.045 | 1.04 | 0.92 | 1.18 | 0.518 |
| Number mobility limitations | 0.98 | 0.89 | 1.07 | 0.598 | 1.02 | 0.97 | 1.06 | 0.436 |
| Number ADL limitations | 1.03 | 0.73 | 1.45 | 0.875 | 1.01 | 0.84 | 1.22 | 0.886 |
| Number IADL limitations | 1.37 | 0.98 | 1.92 | 0.063 | 1.20 | 0.95 | 1.51 | 0.131 |
| Wears a Hearing Aid: (1=Yes 0=No) | NE |  |  |  | 5.00 | 2.26 | 11.09 | 0.000 |
| Vision: (1=Excellent to 5=Poor/Blind) | 1.10 | 0.80 | 1.52 | 0.566 | 1.01 | 0.86 | 1.18 | 0.920 |
| Pain: (1=Yes 0=No) | 1.46 | 0.85 | 2.51 | 0.171 | 0.83 | 0.63 | 1.09 | 0.175 |
| Self-rated Memory:  (1=Excellent to 5=Poor) | 1.20 | 0.84 | 1.72 | 0.309 | 0.92 | 0.78 | 1.08 | 0.316 |
| Number physical symptoms | 1.25 | 1.06 | 1.47 | 0.009 | 0.91 | 0.83 | 1.01 | 0.074 |
| Smoker (yes vs no) | 1.02 | 0.40 | 2.61 | 0.960 | 0.59 | 0.34 | 1.02 | 0.059 |
| Employed (yes vs. no) | 0.58 | 0.27 | 1.25 | 0.165 | 0.84 | 0.61 | 1.16 | 0.286 |
| Finances: (1=Excellent to 5=Poor) | 1.26 | 0.81 | 1.95 | 0.302 | 0.83 | 0.68 | 1.02 | 0.072 |
| Life is close to ideal:  (1=Agree, 2=Neutral; 3=Disagree) | 0.74 | 0.40 | 1.37 | 0.340 | 1.11 | 0.86 | 1.43 | 0.424 |
| Number activities perform | 0.96 | 0.84 | 1.10 | 0.551 | 0.94 | 0.88 | 1.00 | 0.058 |
| Social Support from spouse  (1=A lot to 3=Not at all) | 0.85 | 0.49 | 1.49 | 0.573 | 1.26 | 0.95 | 1.66 | 0.107 |
| Depression: 2012 | 1.07 | 0.97 | 1.19 | 0.168 | 0.99 | 0.94 | 1.04 | 0.709 |
| Depression: 2015 | - | - | - | - | 1.03 | 0.97 | 1.08 | 0.334 |
| Cognition: 2012 | 0.99 | 0.97 | 1.02 | 0.566 | 0.99 | 0.98 | 1.00 | 0.024 |
| Cognition: 2015 | 0.97 | 0.94 | 1.00 | 0.053 | 0.98 | 0.97 | 0.99 | 0.000 |
| Cognition: 2018 | - | - | - | - | 0.99 | 0.95 | 1.03 | 0.523 |

Note: 2012 excluded due to < 10 missing depression in 2012; NE=Not estimable due to small cell size.

| S-Table 4. Logistic Regression Model Results for Death Outcome (1=Deceased, 0=Alive) | | | | |
| --- | --- | --- | --- | --- |
| Variable: | Odds  Ratio | 95% CI | | p-value |
| Female vs male | 0.52 | 0.38 | 0.72 | 0.000 |
| Age in years | 1.11 | 1.08 | 1.14 | 0.000 |
| Educational level | 0.93 | 0.89 | 0.98 | 0.002 |
| Number living children | 1.07 | 1.01 | 1.13 | 0.013 |
| Self-rated health:  (1=Excellent to 5=Poor) | 1.46 | 1.18 | 1.79 | 0.000 |
| Number chronic conditions | 1.26 | 1.09 | 1.44 | 0.001 |
| Number mobility limitations | 1.13 | 1.08 | 1.18 | 0.000 |
| Number ADL limitations | 1.40 | 1.19 | 1.63 | 0.000 |
| Number IADL limitations | 1.79 | 1.47 | 2.18 | 0.000 |
| Wears a Hearing Aid:  (1=Yes 0=No) | 2.81 | 1.00 | 7.89 | 0.050 |
| Vision:  (1=Excellent to 5=Poor/Blind) | 1.26 | 1.04 | 1.52 | 0.018 |
| Pain: (1=Yes 0=No) | 1.04 | 0.76 | 1.42 | 0.818 |
| Self-rated Memory:  (1=Excellent to 5=Poor) | 1.25 | 1.02 | 1.53 | 0.033 |
| Number physical symptoms | 1.14 | 1.04 | 1.27 | 0.008 |
| Smoker (yes vs no) | 1.27 | 0.78 | 2.07 | 0.342 |
| Employed (yes vs. no) | 0.56 | 0.37 | 0.86 | 0.008 |
| Finances:  (1=Excellent to 5=Poor) | 1.41 | 1.09 | 1.83 | 0.009 |
| Life is close to ideal:  (1=Agree, 2=Neutral; 3=Disagree) | 1.05 | 0.77 | 1.43 | 0.752 |
| Number activities perform | 0.84 | 0.77 | 0.91 | 0.000 |
| Social Support from spouse  (1=A lot to 3=Not at all) | 0.82 | 0.60 | 1.13 | 0.233 |
| Depression: 2012 | 1.04 | 0.98 | 1.11 | 0.148 |
| Depression: 2015 | 1.09 | 1.02 | 1.15 | 0.006 |
| Cognition: 2012 | 0.97 | 0.96 | 0.98 | 0.000 |

| S-Table 5. Sensitivity Analysis of Associations between Cognition and Depression (Model 1: Paths from cognition to depression) | | | | | | | | | | |
| --- | --- | --- | --- | --- | --- | --- | --- | --- | --- | --- |
|  | Reported Results | | Complete  Case  Analysis | | Missing Not At Random (MNAR)  Missing set to Mean | | Missing Not At Random (MNAR)  Missing set to  Poorer Outcomes | | Missing Not At Random (MNAR)  Missing set to  Better Outcome | |
|  | β | p-value | β | p-value | β | p-value | β | p-value | β | p-value |
| Hypothesis 1a (actor): |  |  |  |  |  |  |  |  |  |  |
| Cog → Cog | **.503** | < .001 | **.569** | < .001 | **.456** | < .001 | **.481** | < .001 | **.447** | < .001 |
| Hypothesis 2a (partner): |  |  |  |  |  |  |  |  |  |  |
| Cog → Partner Cog | .011 | .545 | -0.016 | .436 | .010 | .516 | .004 | .798 | .013 | .432 |
| Hypothesis 1b (actor): |  |  |  |  |  |  |  |  |  |  |
| Depress → Depress | **.333** | < .001 | **0.378** | < .001 | .**328** | < .001 | **.333** | < .001 | **.324** | < .001 |
| Hypothesis 2b (partner) |  |  |  |  |  |  |  |  |  |  |
| DepressPartner Depress | .034 | .074 | 0.038 | .066 | .032 | .056 | .031 | .069 | .031 | .070 |
| Hypothesis 3 (actor) |  |  |  |  |  |  |  |  |  |  |
| Cog Depress | **-.009** | .011 | **-0.010** | .019 | **-.009** | .005 | **-.013** | < .001 | **-.007** | .023 |
| Hypothesis 4 (partner) |  |  |  |  |  |  |  |  |  |  |
| Cog → Partner Depress | -.002 | .549 | -0.003 | .525 | -.002 | .494 | -.002 | .621 | -.002 | .478 |

Note: Complete case analysis used mean of the seven cognitive tasks that were not missing. For MNAR scenarios poorer outcome assigned is 1 standard deviation above the mean for depression, and one standard deviation below the mean for cognition. Better

outcome assigned is 1 standard deviation below the mean for depression, and one standard deviation above the mean for cognition.

| S-Table 6. Sensitivity of Associations between Cognition and Depression (Model 2: Paths from depression to cognition) | | | | | | | | | | |
| --- | --- | --- | --- | --- | --- | --- | --- | --- | --- | --- |
|  | Reported Results | | Complete  Case  Analysis | | Missing Not At Random (MNAR)  Values set to Mean | | Missing Not At Random (MNAR)  Missing set to  Poorer Outcomes | | Missing Not At Random (MNAR)  Missing set to  Better Outcome | |
|  | β | p-value | β | p-value | β | p-value | β | p-value | β | p-value |
| Hypothesis 1a (actor): |  |  |  |  |  |  |  |  |  |  |
| Cog → Cog | **0.496** | < .001 | **.558** | < .001 | **.449** | < .001 | **.471** | < .001 | **.439** | <.001 |
| Hypothesis 2a (partner): |  |  |  |  |  |  |  |  |  |  |
| Cog → Partner Cog | 0.006 | .738 | -0.024 | .235 | .007 | .630 | .000 | .985 | .010 | .539 |
| Hypothesis 1b (actor): |  |  |  |  |  |  |  |  |  |  |
| Depress → Depress | **0.341** | < .001 | **0.388** | < .001 | **.336** | < .002 | **.344** | < .001 | **.332** | < .001 |
| Hypothesis 2b (partner) |  |  |  |  |  |  |  |  |  |  |
| DepressPartner Depress | **.038** | .041 | **0.046** | .027 | **.035** | .034 | **.035** | .037 | **.034** | .043 |
| Hypothesis 3 (actor) |  |  |  |  |  |  |  |  |  |  |
| Depress Cog | **-0.193** | .030 | **-0.273** | .014 | **-.160** | .053 | **-.204** | .014 | -.141 | .108 |
| Hypothesis 4 (partner) |  |  |  |  |  |  |  |  |  |  |
| Depress → Partner Cog | -0.102 | .228 | -0.203 | 0.059 | -.041 | .585 | -.042 | .585 | -.036 | .648 |

Note: Complete case analysis used mean of the seven cognitive tasks that were not missing. For MNAR scenarios poorer outcome assigned is 1 standard deviation above mean for depression, and one standard deviation below the mean for cognition. MNAR Better outcome assigned is 1 standard deviation below mean for depression, and one standard deviation above the mean for cognition.
